# What works to improve school lunch nutritional quality – legislation or self-audit?

**DOI:** 10.1017/S1368980022000817

**Published:** 2022-07

**Authors:** Emma Patterson, Filip Andersson, Liselotte Schäfer Elinder

**Affiliations:** 1 Department of Global Public Health, Karolinska Institutet, Stockholm 171 77, Sweden; 2 Centre for Epidemiology and Community Medicine, Region Stockholm, Stockholm 104 31, Sweden

**Keywords:** Implementation, Evaluation, Food environment, Instrument, Audit and feedback

## Abstract

**Objective:**

Sweden updated its legislation on universal free school meals in 2011 and nutrition was explicitly mentioned. The current study (i) describes cross-sectional changes in school lunch nutritional quality during the following eight years and (ii) examines if repeated self-auditing, using a fully automated, online tool (School Food Sweden), based on the implementation strategy of audit and feedback, was associated with improvements.

**Design:**

Both repeated cross-sectional and longitudinal design. Factors associated with meeting nutritional criteria were examined using variance weighted least squares regression and logistic regression.

**Setting:**

Sweden.

**Participants:**

Primary schools who self-selected to audit meal quality between March 2012 and July 2019.

**Results:**

Almost half of all (ca 4800) primary schools signed up to use the tool and 1500 audited nutritional quality at least once. Repeated cross-sectional analyses showed the proportion meeting the nutritional criteria increased significantly between 2012/13 (11 %) and 2018/19 (34 %). Longitudinally, each additional audit completed increased the odds of meeting the nutritional criteria by 1·30 (CI 1·20, 1·41), controlling for region and time elapsed since the legislative change. In 774 schools with repeat audits, both number of audits and frequency of accessing feedback predicted meeting the nutritional criteria (OR 2·02, CI 1·23, 3·31), even after adjusting for time since the legislative change and days elapsed since previous audit.

**Conclusions:**

Both legislation and self-audit with automatic feedback appear effective in helping schools to improve school meal quality. Self-audit with feedback may be an effective complement to legislation, or a promising alternative in settings where regulation is not an option.

The WHO recognises the importance of school meals and recommends school meal policies as a way to improve public health^(1)^. While many countries offer school meals, contexts and policies vary greatly^(2–6)^. However, one common issue is a lack of monitoring and evaluation^(5,7,8)^, particularly in high-income countries^(3)^. This in turn limits the evidence base for school meal policies, which in turn can hamper the spread of good or improved practice.

Universal policies and long-standing practices are particularly challenging to evaluate^(5)^, and this is exemplified by Sweden where universal school meals have a long history (see Box 1). Sweden is almost unique in providing school lunches free of charge to all primary school pupils – ages 6–16 – regardless of the economic circumstances of the family, or whether the school is publicly (i.e. municipality) or privately run. Yet according to a recent overview of policies in selected European countries^(5)^, neither Sweden nor Finland, which has a very similar school meal system^(9)^, would have met that review’s core criteria for ‘good practice’, as both countries lack an official system for monitoring and evaluation. In Sweden, new legislation on education came into effect in 2011 explicitly stating for the first time that school meals should be ‘nutritious’ (see Box 1). This provided a new opportunity for a policy evaluation, but none was officially planned. No further clarification of ‘nutritious’ was provided but the School Inspection Agency, who has the task of following up implementation of all aspects of the school law, interpreted it to mean that meals should be in line with Swedish (now Nordic) nutritional recommendations, and that schools should include meals in their systematic routines for quality control^(10)^.

Box 1. Selected major developments in Swedish school lunch policy

Late 19^th^ century: School lunches are provided piecemeal, as a way of counteracting poverty

1946: National policy is introduced to subsidise meals if local authorities chose to provide them

1970s: Implementation of school lunches is now widespread

1998: Education Act 1997 comes into force; school meals to be provided to all, ‘free of charge’

2011: Education Act 2010 comes into force and adds that school meals should be ‘nutritious’

For a more detailed history, see Lundborg *et al.* ^(29)^.

As no official monitoring or evaluation was planned, in 2010 a tool was developed by researchers to allow schools complete a self-assessment (audit) of their school meal situation. One aim of the tool was to build up a database and to evaluate any changes in nutritional quality after the new policy. Another aim was to support schools and municipalities in their attempts to improve overall school meal quality, by providing them with automatic tailored feedback^(11)^. Audit and feedback is an implementation strategy defined as ‘a summary of […] performance over a specified period of time, given […] in a written, electronic or verbal format. The summary may include recommendations for […] action’^(12)^. Self-evaluation is considered a useful tool in the field of school effectiveness^(13)^, and a Cochrane review found evidence for the effectiveness of audit and feedback for improving practice in the healthcare setting^(14)^. However, another Cochrane review of strategies used to enhance implementation of school-based policies or practices – targeting risk factors such as nutrition, physical activity or tobacco – found that audit and feedback was rarely utilised in this setting^(15)^.

Using a pre-post study design, between spring 2011 and spring 2013, the initial effects of the policy were evaluated in a randomly selected, nationally representative, sample of schools. That study found significant but modest improvements in nutritional quality, defined as schools meeting nutritional criteria^(16)^. In the present study, using the same outcome as the previous study, we wanted to examine the effects of the policy over a longer time period, as well as, for the first time, evaluate the effect of repeatedly using the tool. The aims of the current study were therefore to describe the changes in nutritional quality of school lunches offered in Swedish primary schools in the eight years following a change to national legislation (research question [RQ] 1) and to examine if repeated use of a self-audit tool was associated with improvements (RQ2).

## Methods

### The School Food Sweden tool

The development and validation of the tool is described in more detail elsewhere^(11)^. Briefly, a stakeholder group helped identify six important domains of school meal quality – provision/choice, nutritional quality, food safety issues, service and pedagogy, environmental and organisational aspects – and a web-based tool to measure each of these was developed. Following pilot-testing, validation studies and further improvements such as automatic feedback reports, the tool was made freely available for all primary schools in March 2012. The tool consists of two parts: questionnaires – one per domain – plus feedback in the form of a tailored report. The questionnaires are free standing and can be answered in any order; all except the domain ‘provision/choice’ are optional in order to generate a report. The report is a pedagogically designed PDF document, about twenty pages long. It includes summary statistics and clear explanations of why each domain and sub-domain is important; all domains are included, even if not yet completed. The score for each question is shown using a traffic-light colour system to indicate what results are currently good and what could be improved. Schools can contact the administrator of the tool by email or phone if they have questions, but no support, follow-up or other feedback is offered as standard. Schools can use any part of the tool as often as they wish, without limitations. Any member of staff can complete any questionnaire, but more often than not the nutritional quality domain is completed by the school kitchen manager. When the school is ready, they click a button to create and download their tailored feedback.

### Setting, recruitment and study design

Guidelines for school meals are produced by the Swedish Food Agency, a government agency. Guidelines were first issued in 2007; a major revision was published in 2013^(17)^. The guidelines state that meals are expected to meet nutritional recommendations over a four-week period, and include general information about foods to promote, but no standards or rules. In fact, in the most recent revision 2018^(18)^, suggestions of food servings were toned down even more, in order to emphasise the importance of schools themselves having the appropriate knowledge and competence to take a common-sense and holistic approach. This non-prescriptive approach is possible in part because of the long tradition of school meals – even today a school lunch consists of a cooked meal, a salad buffet and crispbread with spread and milk/water. Deep-fried foods have never been a feature, nor have desserts or soft drinks. In the majority of schools, a choice of two or more warm meals is offered, and these days one of those options is very often vegetarian. Food is prepared freshly, either on-site (by municipal or private catering) at a nearby school or at a central municipal kitchen. School cafeterias are common, but while the offering is generally less healthy it is not free of charge, and vending machines are very rare. Pupils are not permitted to bring food from home, but teenagers may generally leave the school premises. Dietary requirements on medical, disability or religious grounds must be accommodated while dietary preferences on ethical or other grounds may be accommodated, if deemed practical. Pupils generally serve themselves and eat in a canteen; teachers are usually present and are encouraged to use the meal as an opportunity to interact with pupils, the ‘pedagogical lunch’^(19)^. The guidelines also emphasise the need to consider other aspects of meal quality, such as the importance of a pleasant meal environment, allowing adequate time to eat, and how school meals have the potential to be integrated with other educational activities^(18)^.

The study population was all primary schools that used the tool between the launch date 29 March 2012 and 31 July 2019. Schools self-select to use the tool, although some public schools may be directed to do so by their municipality. Schools are not invited, and any contact with them prior to sign up is usually indirect – e.g. they have seen the tool mentioned in guidelines, the project manager for the tool has had contact with a municipality or region, or with relevant organisations and government agencies, the tool has been mentioned at relevant meetings/conferences etc. A municipality-level account function that can provide an overview of school account activity and create municipality-level reports was added in 2016.

To examine changes over time (RQ1), we used a repeated cross-sectional design. If a school performed more than one audit of nutritional quality during a school year (defined here as 1 August–31 July), only the most recent was included when reporting that school year’s results at group level. To compare the results of repeated audits (RQ2), we used a longitudinal open cohort design. Due to pilot testing and the pre-post study, some schools had used the tool before the launch date, when automatic feedback was not yet in place. We restricted the analysis in RQ2 to schools that had only ever been exposed to the complete version of the tool, i.e. who had first completed an audit of nutritional quality after the launch date.

### Data collection

#### Nutritional quality

The nutritional quality questionnaire assesses the adequacy of a school’s four-week lunch menu in terms of four nutritional aspects: iron, fibre, vitamin D and fat quality. These four were chosen to focus on nutrients of importance for children that are not easily met^(20)^, including in school lunches^(21,22)^, while keeping the questionnaire as brief as possible. The questionnaire includes questions about the serving frequency of both rich and/or common food sources of these nutrients over a four-week period. All data are self-reported by schools. The answers are scored and compared with validated criteria for the four nutrients^(11)^. If the criteria for all four are met, the school menu is classified as ‘likely to meet nutritional recommendations’, in the current study referred to as ‘meeting nutritional criteria’, the primary outcome. All other results are combined as ‘not meeting nutritional criteria’. Where two audits had been conducted very close together (within 28 d), the later was excluded, on the assumption that this was unlikely to reflect meaningful changes and could signal that the school was testing the effect of alternative answers.

#### Active use of the tool

We extracted data on when and how often the school had performed the audit(s) of nutritional quality, as well as the number of days that had elapsed since any previous audit, and whether feedback (a report with results) had been generated. Some reports are never generated, due to lack of awareness, lack of interest or perhaps technical difficulties and we cannot see if reports have been opened/read. We calculated the proportion of times a school had generated reports and categorised this as *sometimes* (0–50 % of occasions), *mostly* (51–89 % of occasions) and *almost always* (90–100 % of occasions). For public schools, we also noted if and when the municipality had created an account. This variable was included as a proxy for how interested the municipality was in the tool, although this could either signal that schools had support when using it or, conversely, that they were merely under external pressure.

#### School characteristics

Data on schools were extracted from a national database^(23)^, namely: the number of pupils, the owner of the school (municipality or private) and the location of the school. As measures of the school’s socio-economic position, we used the proportion of students with parents with higher education (>12 years of education), as well as the proportion with a foreign background (pupil or both parents born outside Sweden). Occasionally, data were missing, or, if less than 10 pupils in a category, not published. In the latter case, we imputed it as five. School size was categorised into three categories (≤200 pupils, 201–400 pupils and >400 pupils. Geographical location in Sweden was coded as east, south or north, according to one of the definitions used by Statistics Sweden.

### Statistical analysis

For the cross-sectional study (RQ1), the proportion of schools meeting the criteria for nutritional quality each school year was compared and a binary logistic regression was performed to see if school year was a significant predictor. For RQ2, to investigate whether schools with more audits were more likely to meet the nutritional criteria than those with fewer, several analyses were performed. First, we grouped audits from all schools by audit order (i.e. all first audits, all second audits, etc) and compared the proportion of schools meeting the criteria across all groups, calculating the average results and the average change from the preceding audit. Second, as selection bias was a potential concern, i.e. schools that went on to use this tool many times might differ from ones that only used the tool once, we repeated the current analysis, stratifying schools according to the total number of audits performed, to see if the pattern held. Schools with more than nine audits were excluded due to very small numbers (*n* 13, 1 %).

Third, we performed a subgroup analysis that allowed us to control for potential confounders, using variance weighted least squares (VWLS) regression. This model, sometimes referred to as meta-regression, extends simple linear regression to consider the outcome as an estimated quantity that can be averaged, rather than a simple observation. For each subgroup (audits grouped by audit order), the variances of the outcome variable are estimated and assumed independent of the other subgroups. Then the model treats each subgroup as one observation, weighted with the estimated variance. In general, the outcome variable can be seen as an estimate and the explanatory variables as confounders observed at subgroup level that might influence the average of the ‘intervention’ effect. Here, we estimated the continued effect of total number of audits with and without the potential confounders included in the models. The confounders controlled for in the models were distribution of region, proportion of private schools and average size of the schools.

Finally, as the tool consists of two components – an audit component plus a feedback component – we wanted to consider both as independent predictors. Logistic regressions were performed to test if the odds of meeting the nutritional criteria was predicted by a) the number of occasions a school evaluated its nutritional quality or b) the proportion of occasions a school generated its previous feedback. (We first checked there was no evidence of a correlation between number of audits performed and percent of all feedback generated; Spearman’s rho 0·019.) Potential confounding factors in both regression models were audit date (expressed as months since March 2012), school characteristics and, for public schools, whether the municipality had an account by the time of the school’s final audit. Statistical significance was set to a level of 0·05. Analysis was performed in IBM SPSS Statistics for Windows (version 26), except for the variance weighted least squares which was performed in Stata Statistical software (version 16·1).

## Results

### Use of the tool

By July 2019, 2206 primary schools had created an account, corresponding to 45 % of all primary schools in Sweden that year (ca 4800) (Table 1). Additionally, 50 % of the country’s 290 municipalities had created a municipal-level account. During the 7-year period from launch spring 2012 to end of school year 2018/19, 1500 schools audited nutritional quality at least once. These schools came from 223 of the country’s 290 municipalities. In total, 4141 audits of nutritional quality were made during this period; 894 schools (57 %) performed two or more audits. For RQ2, 190 schools were excluded, as they had first used the tool before the report function was available; 1310 schools remained. Schools using the tool were not representative of all schools nationally. They tended to be larger, were more likely to be publicly run, and more likely to be from the eastern region of Sweden (Table 1). This pattern remained relatively stable, making it reasonable to compare trends over time.

**Table 1:** Description of all schools, schools with accounts and schools with completed audits

					Schools that have evaluated nutritional quality			
	All schools*		Schools with accounts		All		Post-launch†	
	*n* 4823		*n* 2206		*n* 1500		*n* 1310	
	*n* §	%	*n*	%	*n*	%	*n*	%
Schools (% of all schools)		100		45·0		31·0		27·1
Municipal schools (%)		82·7		88·4		91·9		91·5
Number of pupils (median)		208		285		300		286
Region (%)								
East		32·7		40·6		44·7		42·3
South		45·7		42·1		37·9		39·5
North		21·5		17·4		17·3		18·2
Municipality has created account (%)‡		n/a	1951	78·2	1378	85·1	1198	77·6
Parents with higher education (%)	4644	54·0		–	1380¶	51·0	1216	50·1
Pupils with foreign background (%)	1153	15·0		–	1085**	13·0	1180	13·0

* Operating in 2017/18.

† Excluding schools which began to use the tool prior to March 2012.

‡ For municipally run schools only.

§ N can vary due to missing data so the *n* for which data are available is given if different from the *n* in the header.

¶ Missing data for higher education: 120 of 1500 (of which 91 due to difficulties locating data, nine lacked data, twenty had less than ten pupils with this characteristic and so data are not made public (imputed as 5)).

** Missing data for foreign background: 415 of 1500 (of which 91 due to difficulties locating data, 50 lacked data, 274 had less than ten pupils with this characteristic and so data are not made public (imputed as 5)).

### Changes over time

Many schools had difficulty meeting the nutritional criteria for school meals (Table 2). However, the cross-sectional results (RQ1) showed the proportion increased significantly with each passing school year, from 11 % in the first full school year of operation 2012/13 to 34 % in 2018/19 (Table 2). Of the four nutrients included in the tool, schools had most difficulty reaching the requirements for vitamin D and fat quality, while requirements for fibre and iron were met by most (data not shown). As schools included in these yearly cross-sectional datasets included both schools performing an audit for the first time and those that were repeat users, we examined if this positive trend was also present among first-time users only, who could not have been affected by previous experience with the tool. No such clear trend was seen, and the variation from year-to-year was high (Table 2).

**Table 2:** Proportion of schools meeting all four nutritional criteria per school year

	ST 2012		2012/13		2013/14		2014/15		2015/16		2016/17		2017/18		2018/19	
	*n*	%	*n*	%	*n*	%	*n*	%	*n*	%	*n*	%	*n*	%	*n*	%
All schools using tool	64		554†		604		519		635		547		464		451	
Meeting nutritional criteria‡	6	9·4	59	10·6	160	26·5*	140	27·0*	181	28·5*	181	33·1*	150	32·3*	153	33·9*
Schools using tool for first time§	55		430		343		123		177		85		53		44	
Meeting nutritional criteria	4	7·3	38	8·8	58	16·9*	24	19·5*	13	7·3	9	10·6	4	7·5	11	25·0*

ST, spring term.

† Includes ninety-four schools participating in the pre/post study from 2011 and 2013 (i.e. not self-selected).

‡ All four nutritional criteria, based on a school’s final audit for that school year.

§ Schools who had completed an audit prior to launch date were excluded.

* Significantly different from reference year 2012/13, the first complete school year: *P* < 0·01.

### Changes following use of the tool

This longitudinal analysis was restricted to the schools that only ever had access to the complete tool, i.e. first used it March 2012 or later (*n* 1310). Over half audited nutritional quality more than once (59 %, *n* 774). The median length of time between all audits was 367 d (inter-quartile range: 267–502 d). For schools with more than one audit, the proportion meeting all four nutritional criteria on the first audit was 24·5 %, while the proportion meeting the criteria at their final (most recent) audit was 31·6 %.

**Fig. 1:**
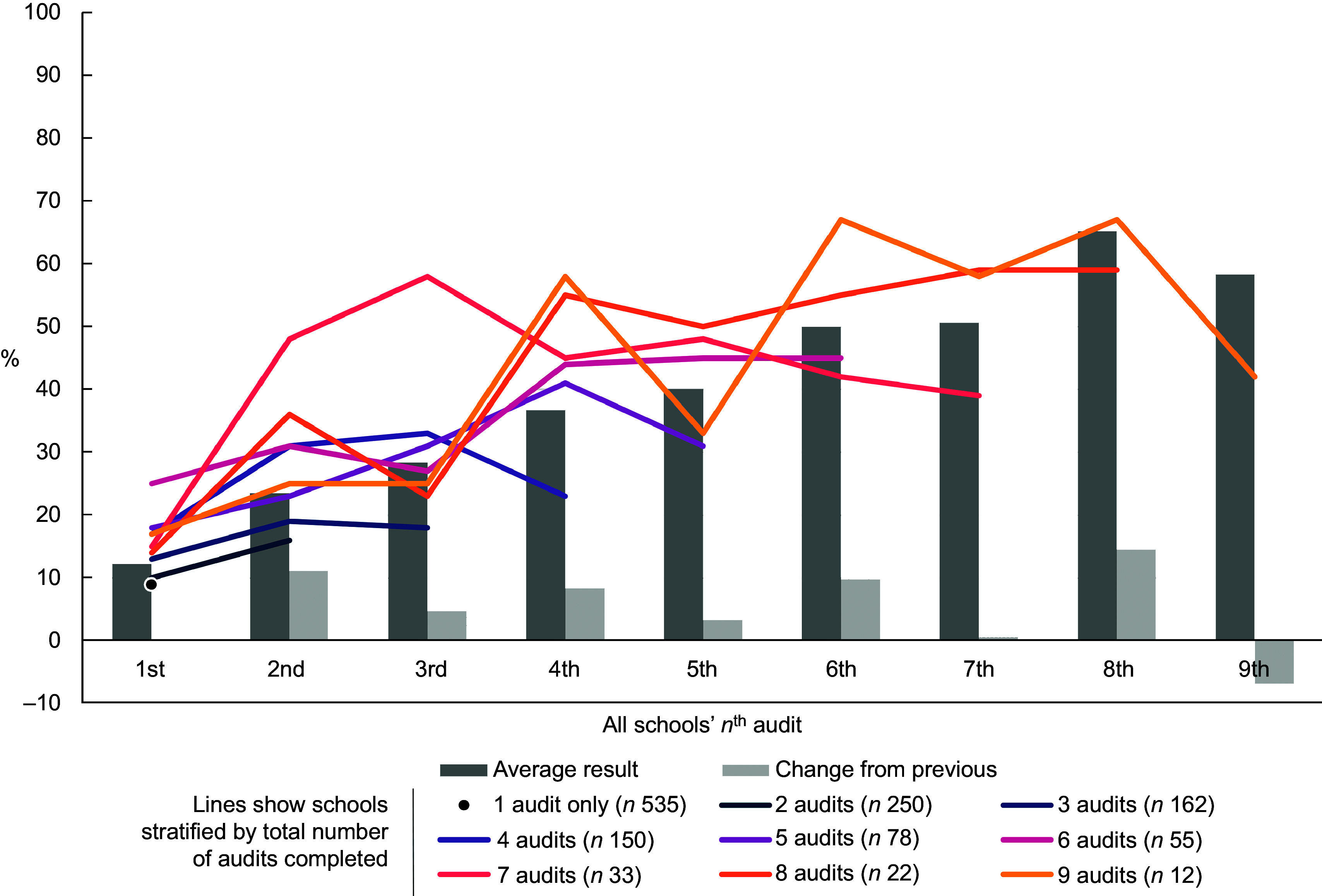
The percentage of schools meeting the nutritional criteria grouped by audit order. Bars show the average results at each audit for all schools combined. Lines show the same data but separately for nine groups of schools: those with only one audit in total (*n* 535 schools), 2 audits in total (*n* 250 schools), etc

To investigate whether schools with more audits were more likely to meet the nutritional criteria than those with fewer (RQ2), several analyses were performed. First, the proportion meeting all criteria at each audit, grouped by audit order, is presented. The bars in Fig. 1 show an overall trend towards improved outcomes by higher audit order. Second, because schools that went on to use the tool repeatedly were more likely to have had better results on the first audit than schools that only ever performed one audit (14·3 % met the criteria *v.* 9·3 %), we stratified schools according to the total number of audits conducted, plotted as lines in Fig. 1. The lines also suggest an overall trend towards improved results, regardless of stratum, although there is a lot of variation, particularly in the strata with most audits due to small numbers.

Third, the results of the variance weighted least squares regression subgroup analysis that allowed adjustment for confounders, showed similar patterns as the results presented in Fig. 1. The estimates showed an increase in average proportion meeting nutritional criteria with increasing number of total audits (data not shown). When comparing the models with and without the potential confounders of school characteristics (distribution of region, proportion of private schools and average size of the schools), no strong indication of confounding effect was observed.

Finally, the results of the logistic regressions show the relationship between a) the total number of audits a school had completed and b) how often (% of times) the school had generated their prior reports, on the likelihood of the school meeting nutritional quality at its final audit. These results are presented in Tables 3 and 4, respectively. In Table 3, with schools with just one audit in total as the reference category, for each increasing number of total audits completed, schools increased their odds of meeting nutritional criteria at their final – most recent – audit by 1·38 (CI 1·30, 1·48, model 1). After controlling for geographical region and audit date, the OR was 1·30 (CI 1·20, 1·41, model 2). When restricting the analysis to 774 schools with repeated uses (and schools with two audits in total as the reference category), results from models 1 and 2 were similar to those for all schools. Model 3 included a variable relevant only to schools with repeated audits, namely the number of days (≥28) that had elapsed since the previous audit. The OR for the final model 3 was 1·26 (CI 1·12, 1·41). Neither the owner of the school, the proportion of pupils with foreign background nor parents with higher education were significant predictors in the models. For municipal schools, we also considered whether the municipality had an account by the time of the school’s final audit, but this was not significant either and was therefore excluded so results could be presented for schools regardless of owner.

**Table 3:** Results of logistic regressions with the ‘total number of audits completed’ as the predictor, showing odds of meeting the nutritional criteria at the final (i.e. most recent) audit

	All schools			All repeat users	
	*n* 1310			*n* 774	
	OR	95 % CI		OR	95 % CI
Model 1	1·38	1·30, 1·48	Model 1*	1·34	1·23, 1·45
Model 2	1·30	1·20, 1·41	Model 2†	1·32	1·20, 1·46
			Model 3‡	1·26	1·12, 1·41

* Unadjusted.

† Model 1 adjusted for region and time since launch.

‡ Model 2 adjusted for days passed since previous audit (only relevant for repeat users).

**Table 4:** Results of logistic regressions with ‘proportion of previous audit results generated’ as the predictor, showing odds of meeting the nutritional criteria at the final (i.e. most recent) audit

	All repeat users	
	*n* 774	
Model 1*	OR	95 % CI
S*ometimes* (0–50 %)	ref.	
*Mostly* (51–89 %)	3·31	1·89, 5·79
*Almost always* (90–100 %)	2·40	1·48, 3·88
Model 2†		
S*ometimes* (0–50 %)	ref.	
*Mostly* (51–89 %)	2·46	1·38, 4·41
*Almost always* (90–100 %)	2·04	1·25, 3·34
Model 3‡		
S*ometimes* (0–50 %)	ref.	
*Mostly* (51–89 %)	1·97	1·09, 3·57
*Almost always* (90–100 %)	2·02	1·23, 3·31

* Model 1: unadjusted.

† Model 2: Model 1 adjusted for region and time since launch.

‡ Model 3: Model 2 adjusted for days passed since previous audit.

In Table 4, with schools that accessed their previous audit results (i.e. generated their report) only *sometimes* as the reference category, schools that accessed their prior results *almost always* were more likely to meet the nutritional criteria: the OR ranged from unadjusted 2·40 (1·48–3·88) to adjusted 2·02 (1·23–3·31, model 3 adjusted as before). The OR in Table 4 were higher than those in Table 3, suggesting accessing feedback was an even stronger predictor of meeting nutritional quality than number of audits.

## Discussion

The findings suggest that both time elapsed since the adoption of a legal requirement for school lunches to be nutritious and repeated use of the School Food Sweden tool, a self-administered audit and feedback tool, exerted an influence on school meal quality in Sweden between 2012 and 2019. Disentangling the two instruments is, however, a challenge due to their universal nature.

Evidence for an effect of the 2011 policy includes the fact that in repeated cross-sectional analyses, the proportion of all schools meeting nutritional criteria increased with each passing year, from 11 % in 2012/13 to 34 % in 2018/19. This extends the results of previous work, where using a pre-post study design, but where no feedback was provided, modest improvements were found in nutritional quality two years after the legislation^(16)^. Legislation is one of the more powerful instruments available to promote behavioural changes^(24)^ and can often give rise to ripple effects – activities and initiatives by other important stakeholders. Some early examples have been described^(16)^, such as the founding of the National Centre for Public Meals (NCPM) at the Swedish Food Agency in 2011. The NCPM overhauled guidelines for school meals in 2013^(17)^ and since then, the guidelines have been disseminated widely. The centre has also undertaken surveys that show that the proportion of municipalities with an official policy document adopted by local politicians for school meals has risen from 45 % in 2011, to 74 % in 2016, and 85 % in 2018^(25)^, and that the vast majority refer to the national guidelines.

However, the repeated cross-sectional analyses showing improvements in quality over time included both schools using the tool for the first time as well as repeat users, so if the tool had an effect it would influence this observation. The proportion of schools meeting nutritional criteria with each passing year did not increase as clearly for schools who were using the tool for the first time, something which would have been expected if time since introduction of the policy was the only factor. Either schools that started using the tool later are different in some way, perhaps with greater needs (a form of selection bias), or the policy effect is smaller than expected, or maybe even waning. Further evidence for the effect of the tool includes the fact that we also saw a dose–response effect, where schools that had used the tool more often had better results than those that used it less often, and furthermore achieved better results when they used both components of the tool – audit together with feedback. In our analysis of factors associated with both improvement in and meeting nutritional criteria, repeated use of the tool stood out as a predictor, even when controlling for other important variables known to be associated with nutritional quality, including time since introduction of the policy. These point to the effectiveness of the tool to improve nutritional quality, rather than the policy alone.

This is not to say that the policy had little effect. As mentioned, the policy led to initiatives and increased attention on school food quality, so the take-up of the tool would likely have been lower if not for the policy. On the other hand, if a policy is not carefully evaluated, it is difficult to be sure of its effects. And without follow-up or consequences for non-compliance, effects may be limited. At school level, the inclusion of school meals in internal quality management systems, as also required by the new law, is quite low. By 2016, only half of schools surveyed by Olsson and Waling had done so^(26)^. Of municipalities with local policy documents, only 58 % had followed these up within the previous three years^(25)^. Poor evaluation and monitoring is a common and persistent problem in the field of school meal policy^(5,7,27)^ and means that good practice and/or lessons learned may be missed. Evaluations of the effects of truly universal meal policies are particularly challenging and are, unsurprisingly, rare^(5)^. A recent systematic review of ‘universal’ school meals – both breakfast and lunch – has been conducted by Cohen *et al.* ^(6)^. They identified studies that predominantly utilised pre-post designs, or where ‘universal’ was limited to a group of schools in a country (not all schools, as in the present study), or in the one case where it was universal – Japan – analysis was cross-sectional. Long-term evaluations are even rarer. For example, the longest follow-up by far in a systematic review of the impact of school food environment policies on actual dietary intakes was 60 months^(28)^. One noteworthy exception is a Swedish study, where economists found that adults who had attended school at a time when free school lunches were becoming widespread in the 50s and 60s and received them during all nine primary school years benefited from a 3 % increase in lifetime earnings, and this effect was greater for those who had come from poorer households^(29)^. That analysis could not take quality into account, and the effects of meals in well-nourished populations are probably less dramatic^(30)^, but the finding that inequalities can be dampened via school meals is relevant even today^(22,31)^.

The implementation strategy of audit and feedback is considered very effective to support change, at least in the healthcare setting^(14)^. It appears to be less commonly used to enhance implementation of school-based health-related policies^(15)^. Evaluations of the effectiveness of such tools and providing feedback remotely – fully automated, without in-person follow-up, as in our study – are rare. One school canteen-based audit and feedback randomised controlled trial has been conducted in Australia^(32)^. Compared with our tool, this was a relatively intensive intervention; the main component being a menu audit, with initial face-to-face contact, and subsequent provision of feedback via a written report and telephone call up to four times over a 12-month period. Although no evidence was found for an improvement in the primary outcomes (proportion of schools with a menu that did not include discouraged foods/beverages, and the proportion where encouraged items made up the majority of the menu), intervention schools offered fewer discouraged items. The intervention has been modified and tested again at scale with positive results^(33)^. In the Netherlands, the Canteen Scan tool^(34)^ has been developed with the aim of assessing compliance with the Dutch Guidelines for Healthier Canteens. This also provides automatic and tailored feedback. In a six-month quasi-experimental controlled trial, improvements in the food environment were noted, but not in pupil purchasing behaviour^(35)^. Again, feedback was not provided remotely, as it was in our case. Otherwise there appear to be few other tools similar to the School Food Sweden tool in terms of function and level of automation, but this may be partly because of difficulties in identifying tools that are not well described in the scientific literature. Two relevant systematic reviews have recently been published. Cupertino *et al.* ^(36)^ identified sixteen instruments (including this one) that have been developed to evaluate school menus. The authors did not assess validity and/or reliability. The majority were not published in English and only one^(37)^ briefly mentioned that software had been developed to automate checklists and provide a PDF of results. O’Halloran *et al.* ^(38)^ reviewed thirty-eight measurement methods which have been used to assess school food environments, of which one-third measured data self-reported at school level. Of these, none of these methods appeared to be designed for use in an ongoing manner, several focused on attitudes and beliefs, and vanishingly few had investigated validity and/or reliability.

### Strengths and limitations

This data set is unique and the long period of time covered is a strength. Although the time period presented here begins after the legislation came into force, the pre- to post-period has been examined separately^(16)^. The tool appears unusual in its degree of automation, requiring little contact with schools, increasing feasibility. The validity and reliability of the tool and the criteria used to assess the nutritional quality have also been described^(11)^. Schools using the tool were not representative of all schools nationally; however, this remained relatively stable, making it reasonable to compare trends over time. The biggest limitations of the study are the self-reported data and the lack of control schools. There is no real incentive for schools not to report accurately as there are no clear consequences for poor results, and the tool is clearly presented as an aid to improvement rather than as a means of control. Still, desirability bias is a common phenomenon and cannot be ruled out. As the policy was national, it was not possible to have control schools that were unexposed to it. As regards schools that were ‘unexposed’ to the tool, we know that they differ with regard to structural factors (e.g. size, owner and region), but we cannot know if the nutritional quality is different. Are schools that decide to use the tool in greater need of help (but maybe less engaged), or do they have better resources (and maybe more engaged), or a mixture? This introduces self-selection bias and unbalanced confounders in estimating the effect of the tool. The effect of using the tool may overstate or understate the true effect. To try to compensate, we explored the question from numerous angles, both at audit level and school level. On the assumption that schools that use it more frequently are more willing and able to improve already from day one, we have, where possible, presented results separately according to frequency of usage. (We found evidence of improvement at all levels of usage.) In effect, we used schools with one audit only and before receiving feedback as ‘control’ – this may in fact be better than using schools that do not use the tool at all, as those with one audit are more likely to be similar to other schools using the tool more often. We therefore believe that the comparison with these groups may actually be less subject to residual confounding. Whether similar improvements are seen in the other five domains of the tool – to give a fuller picture of changes in school meal quality – has not yet been evaluated but is planned.

Long-term evaluations always face the risk of confounding due to other external factors changing over time, likely to be a mixture of positive and negative, which are difficult to account for. For example, we know that challenges for the public meal sector today include replacing the many staff approaching retirement age and the increasing demands on quality, including requests from parents for special dietary requirements^(25)^, changes to budgets and staff training, etc.

## Conclusion

The improvement in nutritional quality of lunches offered in Swedish schools that was first seen two years after the introduction of legislation in 2011 appears to have continued in the subsequent six years. This positive result appears to be at least in part due to repeated use of the School Food Sweden tool. The more schools used the tool, the more likely the lunch menu was to meet nutritional criteria. Self-audit with automatic feedback appears effective in helping schools to improve school meal quality and an essential complement to legislation, or a promising alternative in settings where regulation is not an option.

## References

[1] World Health Organization (2017) Report of the Commission on Ending Childhood Obesity. Implementation Plan: Executive Summary. Geneva, Switzerland: World Health Organisation.

[2] Storcksdieck genannt Bonsmann S (2014) Comprehensive mapping of national school food policies across the European Union plus Norway and Switzerland. Nutr Bull 39, 369–373. 25663818 10.1111/nbu.12109 PMC4314700

[3] World Food Programme (2013) State of School Feeding Worldwide 2013. Rome, Italy: World Food Programme.

[4] Oostindjer M , Aschemann-Witzel J , Wang Q et al. (2017) Are school meals a viable and sustainable tool to improve the healthiness and sustainability of children’s diet and food consumption? A cross-national comparative perspective. Crit Rev Food Sci Nutr 57, 3942–3958. 27712088 10.1080/10408398.2016.1197180

[5] Kovacs VA , Messing S , Sandu P et al. (2020) Improving the food environment in kindergartens and schools: an overview of policies and policy opportunities in Europe. Food Policy 96, 101848.

[6] Cohen JFW , Hecht AA , McLoughlin GM et al. (2021) Universal school meals and associations with student participation, attendance, academic performance, diet quality, food security, and body mass index: a systematic review. Nutrients 13, 911. 33799780 10.3390/nu13030911 PMC8000006

[7] Nelson M & Breda J (2013) School food research: building the evidence base for policy. Public Health Nutr 16, 958–967. 23688714 10.1017/S1368980012005162 PMC10273246

[8] Lucas PJ , Patterson E , Sacks G et al. (2017) Preschool and school meal policies: an overview of what we know about regulation, implementation, and impact on diet in the UK, Sweden, and Australia. Nutrients 9, 736. 28696403 10.3390/nu9070736 PMC5537850

[9] Pellikka K , Manninen M & Taivalmaa S-L (2019) School Meals for All. School Feeding: Investment in Effective Learning – Case Finland. Ministry for Foreign Affairs of Finland and Finnish National Agency for Education; available at https://www.oph.fi/sites/default/files/documents/um_casestudyfinland_schoolfeeding_june2019_netti.pdf (accessed April 2022).

[10] Nordic Council of Ministers (2014) Nordic Nutrition Recommendations 2012 – Integrating Nutrition and Physical Activity. Copenhagen, Denmark: Nordic Council of Ministers.

[11] Patterson E , Quetel AK , Lilja K et al. (2013) Design, testing and validation of an innovative web-based instrument to evaluate school meal quality. Public Health Nutr 16, 1028–1036. 23009762 10.1017/S1368980012004211 PMC10271470

[12] Effective Practice and Organisation of Care (EPOC) EPOC Taxonomy; available at www.epoc.cochrane.org/epoc-taxonomy (accessed April 2022).

[13] Chapman C & Sammons P (2013) School Self-Evaluation for School Improvement: What Works and Why? Reading, UK: CfBT Education Trust.

[14] Ivers N , Jamtvedt G , Flottorp S et al. (2012) Audit and feedback: effects on professional practice and healthcare outcomes. Cochrane Database Syst Rev, Issue 6, CD000259. doi: 10.1002/14651858.CD000259.pub3. 22696318 PMC11338587

[15] Wolfenden L , Nathan NK , Sutherland R et al. (2017) Strategies for enhancing the implementation of school-based policies or practices targeting risk factors for chronic disease. Cochrane Database Syst Rev, Issue 11, CD011677. doi: 10.1002/14651858.CD011677.pub2. 29185627 PMC6486103

[16] Patterson E & Elinder LS (2015) Improvements in school meal quality in Sweden after the introduction of new legislation – a 2-year follow-up. Eur J Public Health 25, 655–660. 25395403 10.1093/eurpub/cku184

[17] Livsmedelsverket [Swedish Food Agency] (2013) Bra mat i skolan [Good Food in School]. Uppsala, Sweden: Swedish Food Agency.

[18] Livsmedelsverket [Swedish Food Agency] (2018) Nationella riktlinjer för måltider i skolan [National Guidelines for Meals in Schools]. Uppsala, Sweden: Swedish Food Agency.

[19] Persson Osowski C , Goranzon H & Fjellstrom C (2013) Teachers’ interaction with children in the school meal situation: the example of pedagogic meals in Sweden. J Nutr Educ Behav 45, 420–427. 23768894 10.1016/j.jneb.2013.02.008

[20] Barbieri HE , Pearson M & Becker W (2006) Riksmaten barn 2003. Livsmedels- och näringsintag bland barn i Sverige [Riksmaten Children 2003. Food and Nutritional Intake of Children in Sweden]. Uppsala, Sweden: Swedish Food Agency.

[21] Persson Osowski C , Lindroos AK , Enghardt Barbieri H et al. (2015) The contribution of school meals to energy and nutrient intake of Swedish children in relation to dietary guidelines. Food Nutr Res 59, 27563. 26522664 10.3402/fnr.v59.27563 PMC4628944

[22] Colombo PE , Patterson E , Elinder LS et al. (2020) The importance of school lunches to the overall dietary intake of children in Sweden: a nationally representative study. Public Health Nutr 23, 1705–1715. 32312356 10.1017/S1368980020000099 PMC7267782

[23] Skolverket [Swedish Agency for Education]. Statistics for preschools, schools and adult further education; available at https://www.skolverket.se/skolutveckling/statistik (accessed April 2022).

[24] Mozaffarian D , Angell SY , Lang T et al. (2018) Role of government policy in nutrition—barriers to and opportunities for healthier eating. BMJ 361, k2426. 29898890 10.1136/bmj.k2426 PMC5997034

[25] Grausne J & Quetel AK (2018) Fakta om offentliga måltider 2018 [Facts about Public Meals 2018]. Uppsala, Sweden: Swedish Food Agency.

[26] Olsson C & Waling M (2016) School meals do not have a given place in Swedish school’s quality management. Health Educ J 75, 961–971.

[27] Chambers S , Boydell N , Ford A et al. (2020) Learning from the implementation of Universal Free School Meals in Scotland using Normalisation Process Theory: lessons for policymakers to engage multiple stakeholders. Food Policy 95, 101936. 33041438 10.1016/j.foodpol.2020.101936 PMC7539368

[28] Micha R , Karageorgou D , Bakogianni I et al. (2018) Effectiveness of school food environment policies on children’s dietary behaviors: a systematic review and meta-analysis. PLoS One 13, e0194555. 29596440 10.1371/journal.pone.0194555 PMC5875768

[29] Lundborg P , Rooth D-O & Alex-Petersen J (2021) Long-term effects of childhood nutrition: evidence from a school lunch reform. Rev Econ Stud 89, 876–908.

[30] Ells LJ , Hillier FC , Shucksmith J et al. (2008) A systematic review of the effect of dietary exposure that could be achieved through normal dietary intake on learning and performance of school-aged children of relevance to UK schools. Br J Nutr 100, 927–936. 18377677 10.1017/S0007114508957998

[31] Kristjansson EA , Robinson V , Petticrew M et al. (2007) School feeding for improving the physical and psychosocial health of disadvantaged elementary school children. Cochrane Database Syst Rev, Issue 1, CD004676. doi: 10.1002/14651858.CD004676.pub2. 17253518

[32] Yoong SL , Nathan N , Wolfenden L et al. (2016) CAFÉ: a multicomponent audit and feedback intervention to improve implementation of healthy food policy in primary school canteens: a randomised controlled trial. Int J Behav Nutr Phys Act 13, 126. 27919261 10.1186/s12966-016-0453-z PMC5139098

[33] Reilly KL , Nathan N , Wiggers J et al. (2018) Scale up of a multi-strategic intervention to increase implementation of a school healthy canteen policy: findings of an intervention trial. BMC Public Health 18, 860. 29996817 10.1186/s12889-018-5786-x PMC6042415

[34] Voedingscentrum [Netherlands Nutrition Centre]. Healthy School Canteen Guidelines; available at https://issuu.com/voedingscentrum/docs/a_healthy_school_canteen/1?ff&e=1222161/44627946 (accessed April 2022).

[35] Evenhuis IJ , Jacobs SM , Vyth EL et al. (2020) The effect of supportive implementation of healthier canteen guidelines on changes in Dutch school canteens and student purchase behaviour. Nutrients 12, 2419. 32806649 10.3390/nu12082419 PMC7468849

[36] Cupertino AF , Maynard DDC , Queiroz FLND et al. (2021) How are school menus evaluated in different countries? A systematic review. Foods 10, 374. 33572214 10.3390/foods10020374 PMC7915021

[37] Martins Rodrigues C , Giordani Bastos L , Stangherlin Cantarelli G et al. (2020) Sanitary, nutritional, and sustainable quality in food services of Brazilian early childhood education schools. Child Youth Serv Rev 113, 104920.

[38] O’Halloran S , Eksteen G , Gebremariam M et al. (2020) Measurement methods used to assess the school food environment: a systematic review. Int J Environ Res Public Health 17, 1623. 32138232 10.3390/ijerph17051623 PMC7084932

